# HDAC1 Silence Promotes Neuroprotective Effects of Human Umbilical Cord-Derived Mesenchymal Stem Cells in a Mouse Model of Traumatic Brain Injury *via* PI3K/AKT Pathway

**DOI:** 10.3389/fncel.2018.00498

**Published:** 2019-01-04

**Authors:** Ling Xu, Qu Xing, Tuanjie Huang, Jiankang Zhou, Tengfei Liu, Yuanbo Cui, Tian Cheng, Yaping Wang, Xinkui Zhou, Bo Yang, Greta Luyuan Yang, Jiewen Zhang, Xingxing Zang, Shanshan Ma, Fangxia Guan

**Affiliations:** ^1^School of Life Sciences, Zhengzhou University, Zhengzhou, China; ^2^Henan Provincial People’s Hospital, Zhengzhou, China; ^3^Translational Medicine Center, Zhengzhou Central Hospital Affiliated to Zhengzhou University, Zhengzhou, China; ^4^The First Affiliated Hospital of Zhengzhou University, Zhengzhou, China; ^5^Stuyvesant High School, New York, NY, United States; ^6^Department of Microbiology and Immunology, Einstein College of Medicine, Bronx, NY, United States

**Keywords:** histone deacetylase 1, human umbilical cord derived mesenchymal stem cells, traumatic brain injury, neuroprotection, PI3K/AKT

## Abstract

Stem cell transplantation is a promising therapy for traumatic brain injury (TBI), but low efficiency of survival and differentiation of transplanted stem cells limits its clinical application. Histone deacetylase 1 (HDAC1) plays important roles in self-renewal of stem cells as well as the recovery of brain disorders. However, little is known about the effects of HDAC1 on the survival and efficacy of human umbilical cord-derived mesenchymal stem cells (hUC-MSCs) *in vivo*. In this study, our results showed that HDAC1 silence promoted hUC-MSCs engraftment in the hippocampus and increased the neuroprotective effects of hUC-MSCs in TBI mouse model, which was accompanied by improved neurological function, enhanced neurogenesis, decreased neural apoptosis, and reduced oxidative stress in the hippocampus. Further mechanistic studies revealed that the expressions of phosphorylated PTEN (p-PTEN), phosphorylated Akt (p-Akt), and phosphorylated GSK-3β (p-GSK-3β) were upregulated. Intriguingly, the neuroprotective effects of hUC-MSCs with HDAC1 silence on behavioral performance of TBI mice was markedly attenuated by LY294002, an inhibitor of the PI3K/AKT pathway. Taken together, our findings suggest that hUC-MSCs transplantation with HDAC1 silence may provide a potential strategy for treating TBI in the future.

## Introduction

Traumatic brain injury (TBI) is a common brain disorder with high mortality and disability (Reis et al., 2015). Despite the considerable advances in the treatment and nursing of patients, effective therapy to attenuate the pathological process of TBI remain to be explored extensively. Emerging evidence shows that mesenchymal stem cells (MSCs) transplantation can improve the neurologic function following TBI (Kota et al., 2016), which imply MSCs-based therapy is promising for TBI. Human umbilical cord-derived MSCs (hUC-MSCs) have high self-renewal ability, and multidirectional differentiation potential (Xu et al., 2012). However, many studies reported that only a small fraction of transplanted MSCs could migrate, survive and differentiate into neural-like cells in the injured area, which limits its clinical application (Juliandi et al., 2010; Kang et al., 2016). Therefore, it is imperative to develop new strategies to overcome these problems in preclinical studies.

Histone acetylation modification is involved in the development of many neurological diseases, such as Alzheimer’s disease, stroke and cerebral ischemia injury (Benito et al., 2015; Park and Sohrabji, 2016), suggesting that acetylation regulation strategy may be a potential therapeutic avenue to alleviate the neurological dysfunction. It has been demonstrated that histone deacetylase 1 (HDAC1) regulates proliferation and neural differentiation of embryonic stem cells, neural stem cells, tumor stem cells and MSCs (Jacob et al., 2014; Jamaladdin et al., 2014; Cai et al., 2018). More notably, HDAC1 plays a neuroprotective role *in vivo* through enhanced histone acetylation (Lebrun-Julien and Suter, 2015). However, most studies focused on the relationship between HDAC1 and stem cell development *in vitro*, and the regulatory effects of HDAC1 on stem cells *in vivo* are poorly known.

In this study, we found that silencing HDAC1 through siRNA could promote the engraftment of hUC-MSCs in the hippocampus and improve the efficacy of hUC-MSCs transplantation in a TBI mouse model as indicated by improved neurological function, enhanced neurogenesis, decreased neural apoptosis, and reduced oxidative stress in the hippocampus; and, the underlying mechanism of these neuroprotective effects of hUC-MSCs with silenced HDAC1 might involve in the activation of PI3K/AKT pathway.

## Materials and Methods

### Isolation, Culture, and Identification of hUC-MSCs

This study was approved by the Ethics Committees of the Zhengzhou University. hUC-MSCs were isolated, cultured, and identified as previously described (Koh et al., 2008; Wang et al., 2016). After washing cord blood with phosphate buffer solution (PBS), the vessels and umbilical cord membrane were removed. The Wharton’s jelly was cut into about 1-cm^3^ pieces and cultured in DMEM (Hyclone, Logan, UT, USA) with 10% (v/v) fetal bovine serum (Hyclone), 100 U/ml penicillin and 100 μg/ml streptomycin in a 37°C incubator with 5% CO_2_, with media replacement every 3 days. The HDAC1 shRNA and HDAC1 silencing lentivirus (siHDAC1) were designed and synthesized in GenePharm (Shanghai, China) by using lentiviral-vector mediated siRNA targeted HDAC1 silenced expression. The sequence of HDAC1 shRNA is 5′-GCCGGUCAUGUCCAAAGUATT-3′. hUC-MSCs at passage 3 (P3) were plated in 96 well plate for 24 h (2 × 10^3^/well). After transfection at a MOI of 10 for 6 h, cells were rinsed by PBS and maintained in fresh F12-DMEM (10% FBS) for 3 days. HDAC1 expression was detected by Western blotting and quantitative real-time polymerase chain reaction (qRT-PCR).

### Animal Model of TBI

This study was approved by the Institutional Animal Care and Use Committee of Zhengzhou University, China. Procedures were conducted in strict accordance with the National Institutes of Health guidelines for the Care and Use of Laboratory Animals. Male C57BL/6 mice (8–12 weeks, 20–25 g) were housed with free access to food and water on a 12-h light/dark cycle in a pathogen-free environment. After anesthesia with 10% chloral hydrate (200 mg/kg), mice were fixed in a stereotaxic frame and the scalp was shaved. Three millimeter craniotomy was performed over the left parietal cortex (1.5 mm to the anterior fontanelle, and 1.5 mm to the sagittal suture), and the exposed dura was kept intact. Modified Feeney’s weight-drop model was performed for the present study onto the exposed intact cranial dura to produce a standardized parietal contusion (weighing 20 g, falling from 20 cm height; Liu et al., 2013). After trauma, the skull hole was closed with bone wax, and the scalp was sutured.

### Experimental Groups

A total of 120 mice subjected to TBI were randomly divided into four groups: vehicle group, MSCs group, MSCs-siHDAC1 group (MSCs transfected with HDAC1 silencing lentivirus), and LY294002 group. Different treated groups were individually injected into a tail vein. Vehicle group was given 100 μL 0.9% saline. Mice in the MSCs group were injected with 1 × 10^6^ hUC-MSCs suspended in 100 μl 0.9% saline. For the MSCs-siHDAC1 group, mice were injected 1 × 10^6^ hUC-MSCs with HDAC1 silence. For the LY294002 group, 25 mg/kg LY294002 were intraperitoneal administrated 30 min before induction of the TBI model and administered 1 × 10^6^ MSCs-siHDAC1. Mice were intravenously injected with hUC-MSCs at 24 h after operation once a day for 3 days. No mouse died and no aberrant cell growth was observed during the study.

### Behavior Tests

#### Modified Neurologic Severity Score (mNSS)

The neurological functional measurement was evaluated by modified neurologic severity score (mNSS) scoring at 1, 3, 7, 14, 21, and 28 days after treatment by two individuals blinded to the experimental groups. According to this score, the higher the mNSS score, the more severe of TBI deficient. Neurological function was graded on a scale of 0–18 (normal score 0; maximal deficit score 18; Cheng et al., 2015).

#### Morris Water Maze Test (MWM)

Morris water maze test (MWM; Chengdu Taimeng Tech. Co. Ltd., China) was used to evaluate the spatial learning and memory ability of mice as described previously (Cui et al., 2017). Briefly, mice were continuously trained twice daily for six consecutive days, then the navigation test and probe trial were carried out on the 7th day. The latency to find the hidden platform for the 60 s was recorded. A video tracking system recorded the latency time, frequency of platform crossover, time in each quadrant, and the speed.

#### Sucrose Preference Test (SPT)

Mice were trained to consume from two 50-ml bottles of 1% sucrose solution for 3 days, then we replaced one bottle of sucrose with water and allowed mice to drink freely from both bottles for 24 h. In the next 3 days, the respective weights of the sucrose solution and water consumed were recorded and refilled each day at the same time in the morning, and mice were allowed to drink freely from both bottles. Sucrose preference was calculated by using the following formula (Tucker et al., 2017): % Sucrose preference = (sucrose intake/total fluid intake) × 100.

#### Forced Swimming Test (FST)

Mice were placed in a large cylinder (22 cm diameter × 25 cm high) filled with water (13.5 cm high) at a temperature of 23–25°C. Mice movements were recorded for 6 min and mice were placed under a heating lamp to dry upon finishing. Immobility period was defined as motionless floating in the water without struggling and we analyzed immobility time during the last 4 min (Watanabe et al., 2013).

#### Tail Suspension Test (TST)

Mice were suspended by adhesive tape placed approximately 2 cm from the tip of their tails fixing upside down on the hook so that the mouse was suspended 17 cm above a horizontal surface. The immobility time was recorded for 6 min (Cheng et al., 2016). Mice were immobile only when they hung wholly and passively motionless.

### Tissue Preparation

At designated time points, mice were anesthetized and perfused intracardially with 0.9% ice-cold saline. Then, the mice were killed and the brain tissues were collected. The brain tissues were incubated in ice-cold 4% paraformaldehyde (PFA) at 4°C overnight, then transferred into a 30% sucrose solution for 72 h, and sectioned on a cryostat (Leica, Germany) to obtain 20 μm coronal sections. The sections were stored at −80°C until further processing.

### Evaluation of Blood-Brain Barrier (BBB) Permeability

Evans blue (EB) extravasation assay was used to evaluate blood-brain barrier (BBB) permeability at 3 days after TBI. Briefly, 2% EB (4 mL/kg) was injected into the tail vein. Animals were anesthetized after 1 h and perfused using saline to remove intravascular EB dye. The brain was removed and homogenized in phosphate-buffered saline. Trichloroacetic acid was then added to the precipitate protein, and the samples were cooled and centrifuged. The resulting supernatant was measured for the absorbance of EB at 620 nm using a spectrophotometer and quantified as microgram of EB per gram of the brain according to a standard curve.

### Lesion Volume

After MWM testing, mice were sacrificed and perfused transcardially with PBS followed by 4% PFA. Brains were removed and assessed for lesion volume with cresyl violet (CV) staining and myelin integrity with luxol fast blue (LFB) staining as we reported (Cheng et al., 2015). Using the Measure Tool on ImageJ (Version 1.44), a blinded investigator calculated hemispheric brain volume. Lesion volume was obtained by subtracting the volume of brain tissue remaining in the left (ipsilateral) hemisphere from that of the right (contralateral) hemisphere and expressed as percent volume lost.

### Propidium Iodide (PI) Staining

Propidium iodide (PI, Sigma-Aldrich Corporation, St. Louis, MO, USA) staining was performed to assess cell death (Li D. et al., 2016). Briefly, PI (10 mg/ml in saline, 0.4 mg/kg) was administered 1 h before killing by intraperitoneal injection in a total volume of not more than 100 μL. All cortical regions of the brain were chosen from 200× cortical fields from within contused cortex. PI-positive cells were quantitated in the contused cortex in three brain sections and photographed under a DMi8 advanced fluorescence microscope (Leica Microsystems, Germany) using excitation/emission wavelength at 568/585 nm. All images were captured at the same exposure times, contrast settings, and intensity for measurement of fluorescence intensity.

### Reactive Oxygen Species (ROS) Staining

The levels of reactive oxygen species (ROS) in the brain were measured by injecting dihydroethidium (HEt), a specific *in situ* marker of superoxide production (Cheng et al., 2016). Two-hundred microliters of 1 mg/ml HEt was intraperitoneal injected and allowed to circulate for 1 h, anesthetized mice were perfused transcardially with PBS and 4% PFA and brains were taken for immunofluorescence. Sections with similar lesion areas were selected, visualized, and photographed under a DMi8 advanced fluorescence microscope (Leica Microsystems, Germany) using excitation/emission filters at 568/585 nm. All images were captured at the same exposure times, contrast settings, and intensity for measurement of fluorescence intensity.

### Enzyme-Linked Immunosorbent Assay (ELISA)

Upon anesthesia, the peripheral blood of anesthetized mice was harvested and the serum was stored at −80°C until processing. The protein expression or secretion of BDNF, NGF, inflammatory factors (IL-4, IL-10, IL-1β, and TNF-α) levels and oxidative stress levels (MDA, SOD, GSH, and GSH-Px) were measured using enzyme-linked immunosorbent assay (ELISA) kits (Tsz Biosciences, Boston, MA, USA) according to the manufacturer’s instructions.

### Quantitative Real-Time Polymerase Chain Reaction (qRT-PCR)

Total RNA of injured cortical tissue from the perilesional area was extracted by using TRIZOL (Invitrogen, Grand Island, NY, USA) according to the manufacturer’s protocol. The mRNA expression of BDNF, NGF, NSE, DCX, and MAP2 was measured by qRT-PCR, which was calculated by the 2^−ΔΔCt^ method as previously described (Wang et al., 2018). GAPDH was used as the internal standard. Experiments were carried out in triplicate. The sequence of primers for qRT-PCR are shown in Table 1.

**Table 1:** Sequence of primers for quantitative real-time polymerase chain reaction (qRT-PCR).

Gene	Primer (5′-3′)
HDAC1	Forward: 5′-CCAGTATTCGATGGCCTGTT-3′
	Reverse: 5′-AGCAAATTGTGAGTCATGCG-3′
BDNF	Forward: 5′-TCATACTTCGGTTGCATGAAGG-3′
	Reverse: 5′-AGACCTCTCGAACCTGCCC-3′
NSE	Forward: 5′-AGCTCAGGTATCTCCGTGGT-3′
	Reverse: 5′-ACCAGCTCCAAGGATTTATTCTCA-3′
MAP2	Forward: 5′-CTGGACATCAGCCTCACTCA-3′
	Reverse: 5′-AATAGGTGCCCTGTGACCTG-3′
NGF	Forward: 5′-TACAGGCAGAACCGTACACAGATAG-3′
	Reverse: 5′-CAGTGGGCTTCAGGGACAGA-3′
DCX	Forward: 5′-TCCAGTCAGCAAAGGTAAGGA-3′
	Reverse: 5′-CCAAGAGAGAACAGCAAACCA-3′
GAPDH	Forward: 5′-GGTGAAGGTCGGTGTGAAC-3′
	Reverse: 5′-CCTTGACTGTGCCGTTGAA-3′

### Immunofluorescence Staining

Immunofluorescence staining was in accord with previous description (Li D. et al., 2016). After being blocked, coronal sections were incubated with anti-human nuclei antibody (MAB1281, 1:100, Millipore, Oxford, UK), myelin basic protein (MBP; 1:200; Santa Cruz Biotechnology, Dallas, TX, USA), Ki67 (1:200, Bioss, Beijing), DCX (1:200, Proteintech, China), or NeuN (1:200, Proteintech, China) at 4°C overnight and then incubated in Cy3/FITC-conjugated anti-mouse/rabbit anti IgG (1:500, Proteintech, China) for 1 h at room temperature, followed by DAPI (1:2,000, Biotech, China) staining for 10 min. The slides were examined with a DMi8 advanced fluorescence microscope (Leica Microsystems, Germany). Positive-cells were counted with ImageJ software.

### Western Blotting

Total protein of injured cortical tissue from the perilesional area was harvested. Western blotting was performed (Yu et al., 2013). Equal amounts of protein were separated by SDS-PAGE and transferred to PVDF membrane (Millipore, USA). The membrane was blocked with 5% nonfat milk for 2 h at room temperature and incubated with primary antibodies respectively directed against Bcl2 (1:500, Proteintech, China), Caspase3 (1:500, Proteintech, China), Cleaved Caspase3 (1:500, Proteintech, China), HDAC1 (1:500; Abcam, Cambridge, England), PTEN (1:500; Sangon Biotech, Shanghai, China), Phospho-PTEN (Ser380/Thr382/Thr383; 1:500; Sangon Biotech, Shanghai, China), AKT-1 (1:500; Sangon Biotech, Shanghai, China), Phospho-AKT1 (Ser473; 1:500; Sangon Biotech, Shanghai, China), GSK-3β (1:500; Proteintech, Wuhan, China), Phospho-GSK-3β (Ser9; 1:500; Cell Signaling Technology, American), or β-actin (1:5,000; Sangon Biotech, Shanghai, China) at 4°C overnight. After the membrane was washed and incubated with horseradish peroxidase-linked secondary antibody (1:3,000; Sangon Biotech, Shanghai, China) for 2 h. The intensity of the resulting protein bands was quantified using ImageJ software.

### Statistical Analysis

Data are presented as mean ± SEM by using SPSS 21.0 statistical analysis software. One-way or two-way analysis of variance (ANOVA) was used to compare multiple groups. Differences between two groups were tested with the LSD- *t*-test. Data shown were representative of at least three independent experiments. A value of *P* < 0.05 was considered to be statistically significant.

## Results

### Immunophenotypic Characteristics and Expression of HDAC1 in hUC-MSCs

**Figure 1:**
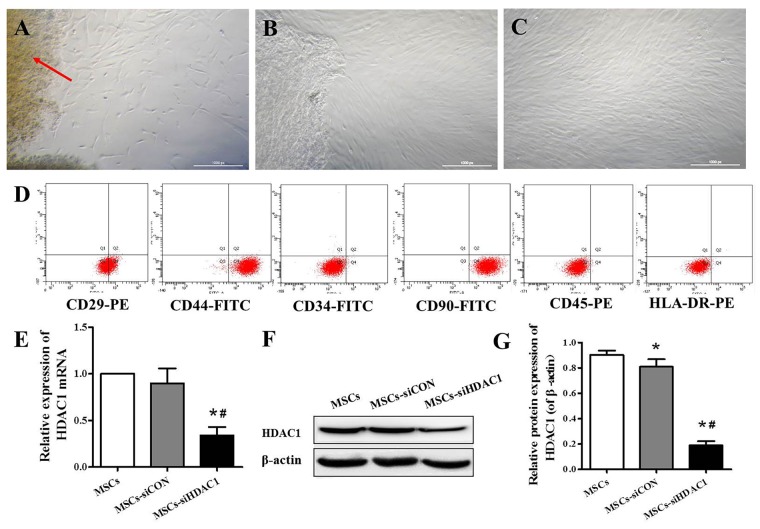
Characterization of human umbilical cord-derived mesenchymal stem cells (hUC-MSCs) and expression of histone deacetylase 1 (HDAC1) in hUC-MSCs. **(A)** Wharton’s jelly tissue pieces (red arrow) were plated and cultured to allow primary hUC-MSCs (P0) to grow out. **(B)** MSCs after 2 weeks of primary culture (P0). **(C)** P3 hUC-MSCs, Scale bar = 100 μm. **(D)** Immunophenotypic characterization of hUC-MSCs by flow cytometry. **(E)** Relative HDAC1 mRNA expression by quantitative real-time-PCR (qRT-PCR). **(F)** Representative expression of HDAC1 by Western blotting and **(G)** densitometric analysis of HDAC1 protein. Data are presented as mean ± SEM. *p < 0.05 vs. MSCs, ^#^*p* < 0.05 vs. MSCs-siCON (MSCs transfected with silencing lentivirus control).

hUC-MSCs derived from Wharton’s jelly, exhibited a fibroblast-like appearance or spindle-shaped morphology at passage 3 (Figures 1A–C). Flow cytometry analysis showed that hUC-MSCs were positive for CD29 (99.9%), CD44 (99.1%), and CD90 (99.8%), but negative for CD34 (0.8%), CD45 (1.6%), and HLA-DR (0.2%; Figure 1D). To investigate the effects of HDAC1 silence to hUC-MSCs, we generated HDAC1 silenced cells by lentivirus transfection. After transfection at a MOI of 10 for 6 h, the expression of HDAC1 in mRNA and protein level was significantly decreased by about 70%–80% in the MSCs-siHDAC1 group when compared with the MSCs group (Figures 1E–G, *p* < 0.05).

### HDAC1 Silence Enhanced the Survival and Migration of hUC-MSCs in the Hippocampus of TBI Mice

**Figure 2:**
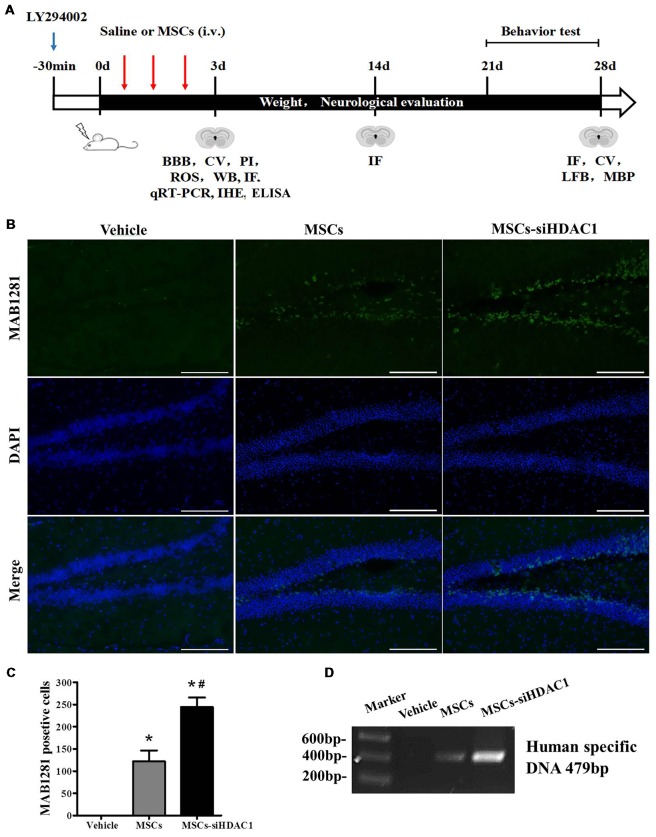
HDAC1 silence enhanced the engraftment of hUC-MSCs in the hippocampus of traumatic brain injury (TBI) mice. **(A)** Overview of experimental design and timeline for experiment. **(B)** Representative immunofluorescent images. **(C)** Quantification analysis of MAB1281^+^ (green) cells in the dentate gyrus of hippocampus, Scale bar = 200 μm. **(D)** Polymerase chain reaction (PCR) results of the human-specific DNA in the hippocampus of TBI mouse. Data were from three mice of each group and three slides of each mouse. Data are presented as mean ± SEM. *p < 0.05 vs. Vehicle, ^#^*p* < 0.05 vs. MSCs.

The complete experimental protocol and timeline were summarized in Figure 2A. Previous studies have shown that TBI-induced secondary damage is sufficient to allow MSCs to across the BBB (Cerri et al., 2015). So, the presence of exogenous hUC-MSCs in the hippocampus was measured by using MAB1281 staining (human nuclei antibody) and PCR for human specific DNA. As shown in Figures 2B,C, the number of MAB1281^+^ cells in the MSCs-siHDAC1 group was significantly higher than that in the MSCs group (*p* < 0.05) and Vehicle group (*p* < 0.05) at 7 days after hUC-MSCs transplantation respectively. PCR result was in accordance with immunofluorescence staining with more human DNA in the MSCs-siHDAC1 group (Figure 2D). These results indicate that HDAC1 silence enhances the survival and migration of hUC-MSCs in the hippocampus of TBI mice after transplantation via tail vein.

### HDAC1-Silenced MSCs Decreased BBB Permeability, Reduced Lesion Volume and Improved Neural Function Recovery After TBI

**Figure 3:**
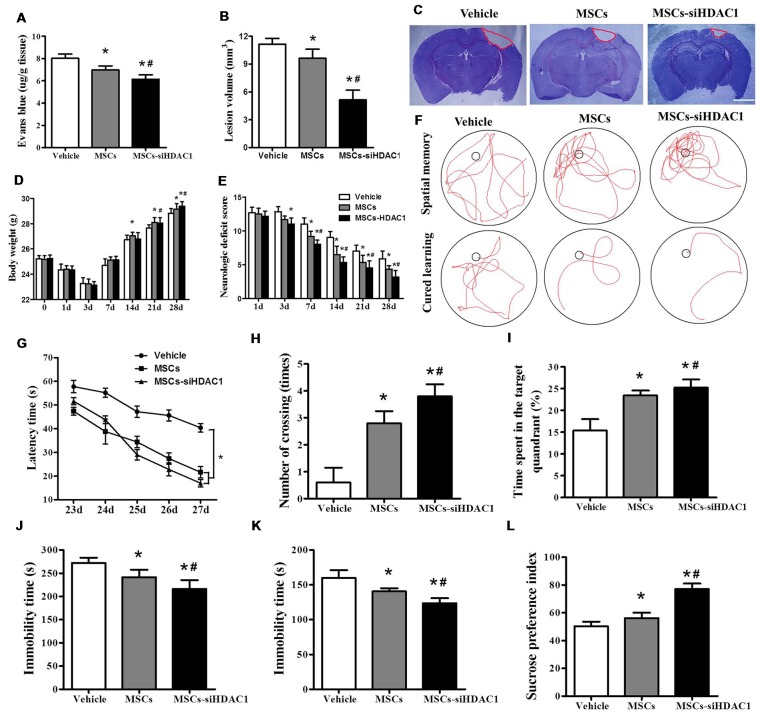
HDAC1-silenced MSCs rescued impaired neural function after TBI. **(A)** Quantitative analysis of extravasated evans blue (EB) dye in the ipsilateral cerebral hemisphere tissue of mice at 3 days after TBI. **(B)** Quantification of lesion volume and **(C)** representative images of cresyl violet (CV) staining, Scale bar = 1 mm. **(D)** Body weight, **(E)** modified neurologic severity score (mNSS) scores. **(F)** Representative tracings from the Morris water maze (MWM) test of the mice. Escape latency **(G)**, platform crossing numbers **(H)**, and time in the target quadrant **(I)** were measured using MWM test. **(J)** Forced swim test (FST), **(K)** tail suspension test (TST), **(L)** Sucrose preference test (SPT). The data were collected from 15 mice in each group and are presented as mean ± SEM. *p < 0.05 vs. Vehicle, ^#^*p* < 0.05 vs. MSCs.

EB dye cannot pass through an intact BBB; however, following TBI, EB easily permeates a compromised BBB (Cerri et al., 2015; Chen et al., 2018). As shown in Figure 3A, MSCs-siHDAC1 group attenuated TBI-induced EB leakage in the ipsilateral hemisphere compared with the vehicle group and MSCs group at day 3-post TBI *(p* < 0.05, Figure 3A). In addition, the lesion volume was significantly reduced in MSCs-siHDAC1 group and MSCs group than vehicle group (*p* < 0.05, Figures 3B,C). From 14 days after TBI, body weight recovered in all treatment groups. Body weight was highest in the MSCs-siHDAC1 group, but least in the vehicle group compared with the other groups (*p* < 0.05, Figure 3D).

The neurological recovery was analyzed by mNSS (*p* < 0.05, Figure 3E), MWM (Figures 3F–I) and the depressive-like behaviors by sucrose preference test (SPT), forced swimming test (FST) and tail suspension test (TST; Figures 3J–L). As expected, the vehicle-infused TBI mice exhibited significant impairments in mNSS and MWM test. However, the mNSS scores were significantly lower in MSCs-siHDAC1 groups on the 3rd to 28th day compared with that of Vehicle and MSCs group (*p* < 0.05, Figure 3E). In the MWM test for evaluating spatial learning and memory ability, MSCs-siHDAC1 transplanted mice performed significantly better than other groups (Figure 3F), as indicated by significantly shorter latency, more crossing numbers and a higher proportion of time spent in the target quadrant (*p* < 0.05, Figures 3G–I).

Additionally, MSCs-siHDAC1 treatment significantly decreased the immobility time of FST and TST compared with those in MSCs and Vehicle treated mice (*p* < 0.05, Figures 3J,K). Similarly, the sucrose preference index was higher in MSCs-siHDAC1 groups compared with those in MSCs groups (*p* < 0.05, Figure 3L). These results indicate that HDAC1-silenced MSCs alleviate BBB permeability and lesion volume, and improve neural function of TBI mice.

### HDAC1-Silenced MSCs Attenuated Oxidative Stress and Neuroinflammation of TBI Mice

**Figure 4:**
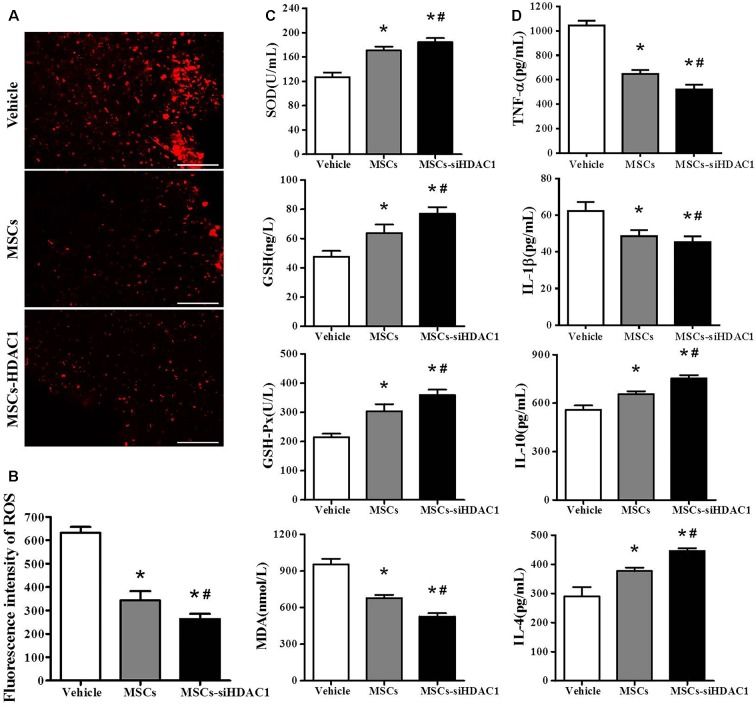
HDAC1-silenced MSCs transplantation attenuated oxidative stress and neuroinflammation in the hippocampus of TBI mice. **(A)** Representative HEt staining on brain sections of different groups at 3 days after TBI. HEt levels in injured cortex. Scale bars = 100 μm. **(B)** Quantification analysis of HEt fluorescence intensity. **(C)** Enzyme-linked immunosorbent assay (ELISA) analysis of SOD, GSH, GSH-Px and MDA at 3 day after TBI. **(D)** ELISA analysis of TNF-α, IL-1β, IL-10, and IL-4 at 3 day after TBI. Data are presented as mean ± SEM. *p < 0.05 vs. Vehicle, ^#^*p* < 0.05 vs. MSCs.

Previous studies demonstrated that TBI resulted in substantial oxidative stress and neuroinflammatory reaction in the injured sites (Angeloni et al., 2015; Corrigan et al., 2016; Russo and McGavern, 2016). So, we assessed the oxidative stress by HEt staining and ELISA. After mice were injected with Het, ROS was observed as red fluorescence signal around the lesion at 3-day post-TBI (Figure 4A). However, the MSCs-siHDAC1 transplanted mice exhibited less ROS production than MSCs group and vehicle groups (Figure 4B, *p* < 0.05). The activity of SOD, GSH, and GSH-Px in the MSCs-siHDAC1 group was significantly higher than those of MSCs and Vehicle group, whereas the level of MDA was lower (Figure 4C, *p* < 0.05). As shown in Figure 4D, the expression level of IL-1β and TNF-α were down-regulated, while, IL-4 and IL-10 were up-regulated in the MSCs-siHDAC1 group (Figure 4D, *p* < 0.05). Our findings suggest that HDAC1-silenced MSCs reduce oxidative stress and neuroinflammation of TBI mice.

### HDAC1-Silenced MSCs Alleviated Myelin Loss and Reduced Cell Death

**Figure 5:**
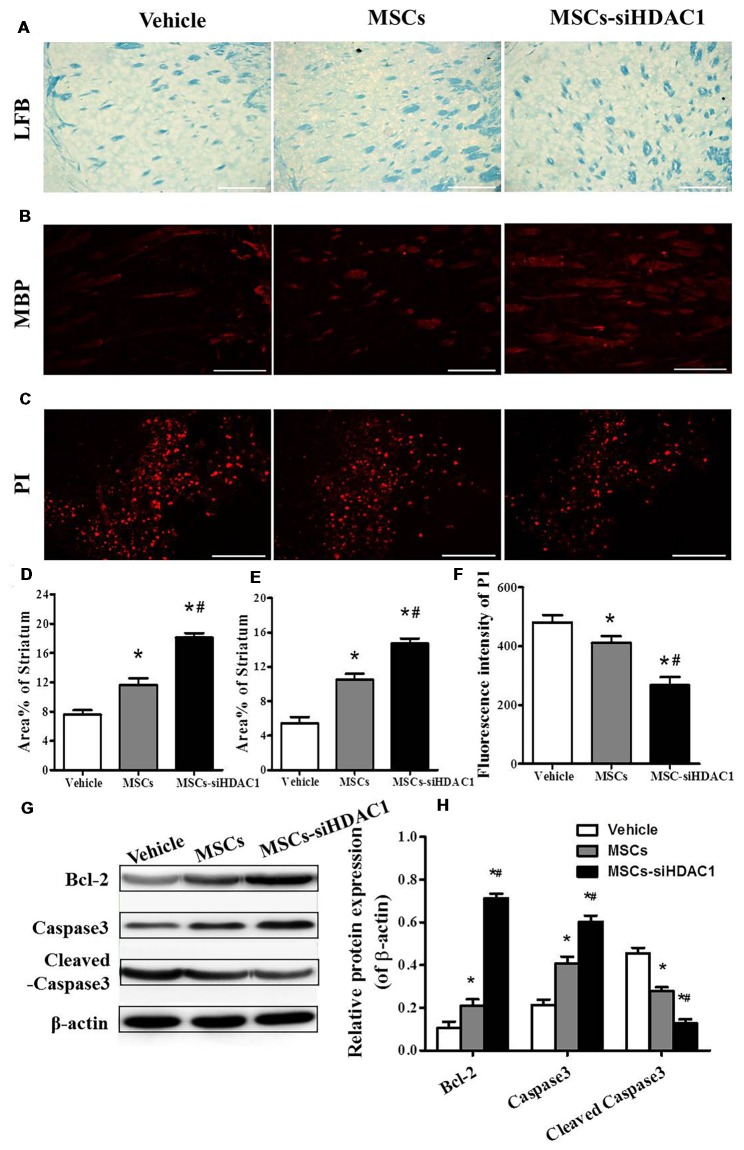
HDAC1-silenced MSCs alleviated white matter injury and reduced cell death after TBI. **(A)** Representative images of luxol fast blue (LFB) staining. Scale bars = 100 μm. **(B)** Myelin basic protein (MBP) staining (red). Scale bars = 200 μm. **(C)** Representative propidium iodide (PI) staining (red) at 3 days after TBI. Scale bars = 100 μm. **(D)** Average area of LFB at 28 days after TBI. **(E)** Average area of MBP. **(F)** Quantitative analysis of PI fluorescence intensity in the injured cortex. **(G)** Western blotting and **(H)** densitometry measurement of Bcl-2, Caspase 3, and Cleaved caspase 3 in the lesion boundary zone of each group at 3 days post-injury. Data are presented as mean ± SEM. *p < 0.05 vs. Vehicle, ^#^*p* < 0.05 vs. MSCs.

It is well known that TBI causes demyelination and neuron death (Gao et al., 2006; Mierzwa et al., 2015). On day 28 after TBI, LFB staining (Figure 5A) and MBP staining (Figure 5B) were used to label normal myelin in the section. Both LFB and MBP staining procedures showed that the percentage of normal-appearing myelin in the MSCs-siHDAC1 group was significantly higher than that in the Vehicle group and MSCs group respectively (*p* < 0.05, Figures 5A,B,D,E). At 3 days after TBI, the cell death in the injured site was measured by PI staining. As shown in Figures 5C,F, MSCs-siHDAC1 decreased PI fluorescence intensity compared with that in the Vehicle group and MSCs group (*p* < 0.05), which was further confirmed by the up-regulated expression of Bcl-2 and Caspase3, while down-regulated of cleaved caspase3 in the hippocampus (*p* < 0.05, Figures 5G,H).

### HDAC1 Silenced MSCs Enhanced the Cell Proliferation Neurogenesis

**Figure 6:**
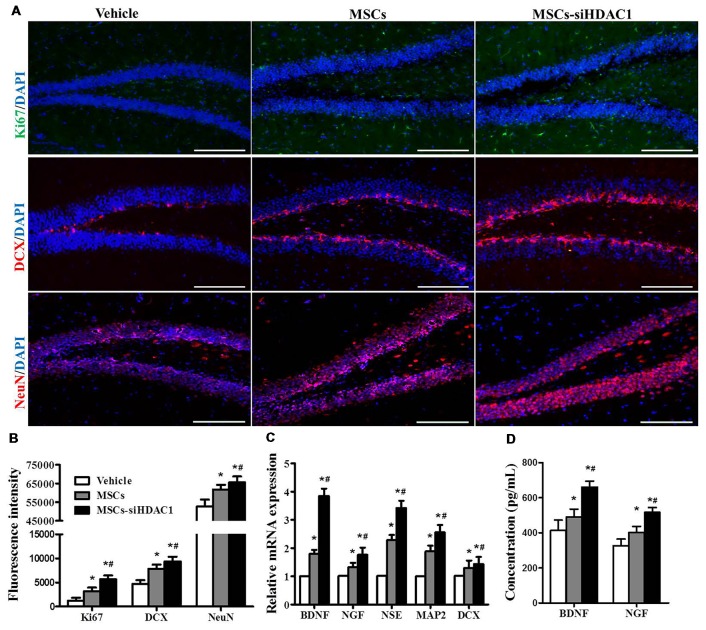
HDAC1-silenced MSCs increased neurogenesis in the hippocampus of TBI mice. **(A)** Representative immunofluorescent images. **(B)** Quantification analysis of fluorescence intensity indicated that Ki67^+^ (green), DCX^+^ (red), and NeuN^+^ (Red) cells in the dentate gyrus of hippocampus in each field, Scale bar = 200 μm. **(C)** Expression of BDNF, NGF, NSE, MAP2 and DCX by qRT-PCR. **(D)** ELISA analysis of BNDF and NGF levels at 28 day after TBI. Data were from three mice of each groups and are presented as mean ± SEM. *p < 0.05 vs. Vehicle, ^#^*p* < 0.05 vs. MSCs.

To further investigate the contribution of MSCs-siHDAC1 to hippocampal neurogenesis, immunofluorescence staining of Ki67 (Acosta et al., 2017; Nuclear proliferation marker), DCX (immature neuronal marker) and NeuN (mature neuronal marker) was performed (Blaya et al., 2015; Neuberger et al., 2017). As shown in Figures 6A,B, MSCs-siHDAC1 group displayed increased fluorescence intensity of Ki67, DCX and NeuN, which represented greater number of Ki67^+^, DCX^+^ and NeuN^+^ cells, compared to those in MSCs group and Vehicle group respectively (*p* < 0.05). These results were confirmed by the enhanced mRNA expression of NSE, MAP2, and DCX in the hippocampus (*p* < 0.05, Figure 6C). Furthermore, compared with Vehicle group, MSCs and MSCs-siHDAC1 obviously increased BDNF and NGF production, which were detected by qRT-PCR and ELISA (*p* < 0.05, Figures 6C,D). So, our findings indicate that MSCs-siHDAC1 treatment enhances hippocampal neurogenesis in TBI mice.

### HDAC1 Silenced MSCs Activated the PI3K/AKT Pathway in Hippocampus of TBI Mice

**Figure 7:**
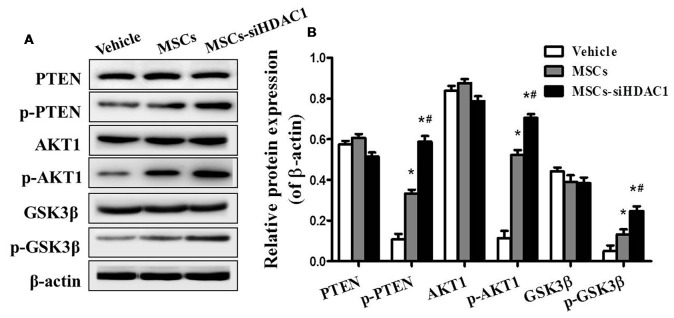
HDAC1-silenced MSCs modulated the protein expression of PI3K/Akt pathway in hippocampus. **(A)** Representative Western blot bands of PTEN, phosphorylated PTEN (p-PTEN), AKT1, phosphorylated AKT1 (p-AKT1), GSK-3β, and phosphorylated GSK-3β (p-GSK-3β) in each group. **(B)** Quantitative protein levels were normalized with β-actin. Data are presented as mean ± SEM. *p < 0.05 vs. Vehicle, ^#^*p* < 0.05 vs. MSCs.

To further study the mechanism underlying the neuroprotective effects of MSCs-siHDAC1 in TBI mice, western blotting was used to examine the protein expression of the PI3K/AKT pathway in the hippocampus at 28 days after TBI. As shown in Figure 7, the expression levels of phosphorylated PTEN (p-PTEN), p-AKT and p-GSK-3β were significantly elevated in the hippocampus of TBI mice after infusion with MSCs and MSCs-siHDAC1 (*p* < 0.05), whereas the total PTEN, AKT and GSK-3β expression remained unchanged (*p* > 0.05, Figure 7B). To conclude, MSCs and MSCs-siHAC1 both activate PI3K/AKT signaling pathway.

### Inhibition of PI3K/AKT Attenuated the Neuroprotective Effect of HDAC1 Silenced MSCs on TBI Mice

**Figure 8:**
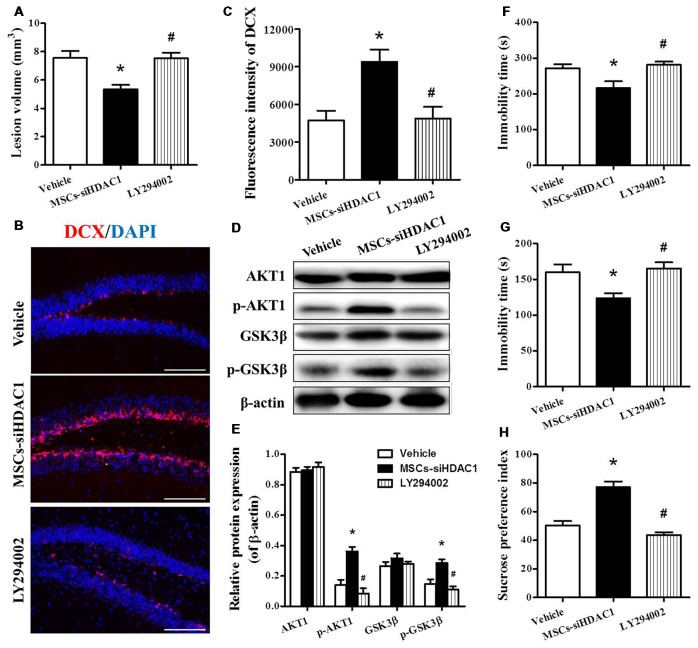
LY294002 abolished the effects of HDAC1-silenced MSCs on neurologic function and the PI3K/AKT pathway of TBI mice. **(A)** Quantification of lesion volume. **(B)** Representative immunofluorescent images. **(C)** Quantification analysis of fluorescence intensity indicated that DCX^+^ (red) cells in the dentate gyrus of the hippocampus in each field, Scale bar = 200 μm. **(D)** Representative Western blot and **(E)** densitometry measurement of AKT1, p-AKT1, GSK-3β, and p-GSK-3β in each group at 3 days post-TBI, **(F)** TST, **(G)** FST, **(H)** SPT. Data are presented as mean ± SEM. *p < 0.05 vs. Vehicle, ^#^*p* < 0.05 vs. MSCs-siHDAC1.

In order to clarify whether MSCs-siHDAC1 exerts a neuroprotective effect by activation of PI3K/AKT signaling, LY294002, a specific inhibitor of the PI3K/AKT pathway (Wen et al., 2018), was injected into mice before TBI and stem cell transplantation. As expected, Western blotting revealed that the protein expression of p-AKT and p-GSK-3β were markedly decreased following co-treatment with LY294002 and MSCs-siHDAC1 (*p* < 0.05, Figures 8D,E) compared with the MSCs-siHDAC1 treated TBI mice. In addition, LY294002 significantly attenuated the MSCs-siHDAC1 induced lesion volume (Figure 8A), neurogenesis (Figures 8B,C) and neurologic function improvement, which appeared as significantly increased immobility time of TST (Figure 8F) and FST (Figure 8G), and decreased sucrose preference index (Figure 8H; *p* < 0.05). Thus, MSCs-siHDAC1 promoted neurologic function recovery of TBI mice by activating the PI3K/AKT pathway.

## Discussion

TBI causes extensive neurologic disability and mortality for individuals worldwide. Currently, no available drug is effective for clinical treatment of TBI. In this study, we report that MSC transplantation with HDAC1 silencing provide neuroprotection in a mouse model of TBI, and this neuroprotective effects is likely due to the activation of PI3K/AKT signaling pathway.

Acetylation/deacetylation of histones is an important mechanism to regulate gene expression and chromatin remodeling (Qureshi and Mehler, 2014). Previous studies have revealed that HDAC inhibition provides a statistically significant protection in Alzheimer’s disease, cerebral ischemia or ischemia/reperfusion model (Shein and Shohami, 2011; Ganai et al., 2016). Furthermore, HDAC1 regulates stem cell proliferation and neural differentiation *in vitro* (Cho and Cavalli, 2014; Lv et al., 2014). The expression of HDAC1 in neurons is surprisingly lower than that in undifferentiated stem cells (Jacob et al., 2014). Our present study supports the view that HDAC1 silencing could promote the migration and neural differentiation of hUC-MSCs in TBI.

TBI patients often suffer from motor and sensory deficits, cognitive impairments, and neuropsychiatric symptoms such as depression and anxiety (Malkesman et al., 2013). MWM is widely used for evaluating learning and memory abilities (Cui et al., 2017). TST, FST, and SPT are widely used to evaluate the depression degree of animals (Watanabe et al., 2013; Cheng et al., 2016). In the current study, our behavioral results showed that Vehicle treated TBI mice exhibited significant motor and cognitive impairments, but these dysfunctions were remarkably attenuated by MSCs and MSCs-siHDAC1 transplantation, following by lower mNSS scores and immobility time, better cognitive capacity, and higher sucrose preference index, indicating that MSCs, especially HDAC1-silenced MSCs transplantation can reduce the depression s and improve the neurologic function of TBI mice.

TBI markedly disturbed the integrity of the BBB and increased extravasation of EB dye (Li H. et al., 2016). Recent evidence indicates that the intravenously transplanted stem cells cross the BBB, migrate to the brain and improve the cognition in AD mice (Xie et al., 2016; Wang et al., 2018). Our results showed that the human cells and human-specific DNA were presented in the hippocampus of TBI mice about 3 days after MSCs transplantation, which could attenuate TBI-induced EB leakage in the ipsilateral hemisphere (Li H. et al., 2016).

TBI results in oxidative stress and immune activation (Corrigan et al., 2016; Russo and McGavern, 2016). When the rapid accumulation of ROS exceeds the capacity of antioxidant system scavenging, this oxidative stress eventually lead to neuronal cell death (Angeloni et al., 2015). In our study, we found that both MSCs and MSCs-siHDAC1 treatment led to a significant decrease of myelin loss, ROS production and MDA level, and an increased tendency of SOD, GSH, and GSH-Px as well as changes of neuroinflammatory markers including TNF-α, IL-1β, IL-4 and IL-10 when compared with the vehicle group respectively. But, MSCs-siHDAC1 treatment showed better effects than MSCs treatment. These findings implied that MSCs alleviated myelin loss, oxidative stress and neuroinflammation of TBI mice might though HDAC1.

Cell death, neurodegeneration and decreased hippocampal neurogenesis occur after TBI secondary brain injury (Reis et al., 2015). Our data showed that MSCs-siHDAC1 alleviated white matter injury and reduced cell death after TBI. Furthermore, transplanted MSCs-siHDAC1 contributed to functional recovery of TBI, which suggested that several mechanisms may be involved in this process. MSCs-siHDAC1 administration may provide a more favorable microenvironment for the activation of neurogenesis. The expression of Ki67, DCX and NeuN in the MSCs-siHDAC1 group was significantly higher than those in MSCs and Vehicle groups, which accompanied by increased expression of BDNF, NGF, NSE, MAP2, and DCX in the hippocampus. Therefore, MSCs-siHDAC1 transplantation may inhibit cell death and promote neurogenesis in the hippocampus of TBI mice.

PI3K/AKT signaling pathway exerts powerful effects on neuronal survival after injury and plays an important role in the neuroprotection, neuronal apoptosis and neurogenesis (Backman et al., 2001; Park et al., 2008; van Diepen and Eickholt, 2008). Meanwhile, some reports have verified that HDAC inhibition modulates the PTEN/PI3K/AKT axis to combat TBI and neurological diseases characterized by white matter as well as gray matter destruction, such as stroke and neurodegenerative disorders (Liu et al., 2012; Wang et al., 2015). PTEN is a negative regulator of PI3K/AKT signal transduction (Backman et al., 2001; Park et al., 2008; van Diepen and Eickholt, 2008). Inhibition of PTEN activity is currently seen as a persuasive target for increasing regenerative capacities of neurons affected in degenerative conditions, or following an injury to the nervous system (Park et al., 2008). In the early stage after TBI, inhibition of PTEN improved neurological function recovery by decreasing BBB permeability and apoptosis (Wang et al., 2015). Previous studies indicated that HDAC inhibition promoted the cytosolic retention of GSK3β (Zhang et al., 2015), where it may be more likely to inactivate cytoplasmic PTEN (Wang et al., 2015). In this study, we found that phosphorylation of PTEN, AKT, and GSK-3β was significantly elevated in the hippocampus of TBI mice after MSCs and MSCs-siHDAC1 transplantation. Interestingly, these MSCs-siHDAC1-induced changes of the expression of p-PTEN, p-AKT, and p-GSK3β along with recovery neurologic function in TBI mice were markedly attenuated by LY294002. Thus, our data suggest that improved neurological function, enhanced neurogenesis, alleviated neural apoptosis and oxidative stress might be mediated by PI3K/AKT pathway.

## Conclusion

HDAC1 silencing promotes hUC-MSCs engraftment in the hippocampus and enhances the efficacy of hUC-MSCs in a TBI model by improving neurological function, enhancing neurogenesis and alleviating neural apoptosis and oxidative stress in the hippocampus. The mechanisms underlying these neuroprotective effects involve in the activation of PI3K/AKT signaling pathway. In conclusion, HDAC1 silenced hUC-MSCs transplantation could provide an effective therapy for TBI.

## Author Contributions

SM and FG conceived and designed the experiments, and revised the final version of the article. BY and TC participated in designing and guiding the experiments. JZha and XZa provided experimental guidance and technical support. LX and QX performed the experiments. LX, QX and TH analyzed the data. SM, LX, YC and FG wrote the manuscript. JZho, TL, YW, XZh and GY prepared reagents and materials and performed part of the experiments. All authors reviewed the manuscript prior to submission.

## Conflict of Interest Statement

The authors declare that the research was conducted in the absence of any commercial or financial relationships that could be construed as a potential conflict of interest.

## Abbreviations

TBI: traumatic brain injury
HDAC1: histone deacetylase 1
hUC-MSCs: human umbilical cord-derived mesenchymal stem cells
BBB: blood-brain barrier
PI: propidium iodide
MWM: Morris water maze test
FST: forced swim test
NOR: novel object recognition
ROS: reactive oxygen species
TST: tail suspension test
SPT: sucrose preference test.
