# Corrigendum: HDAC1 Silence Promotes Neuroprotective Effects of Human Umbilical Cord-Derived Mesenchymal Stem Cells in a Mouse Model of Traumatic Brain Injury *via* PI3K/AKT Pathway

**DOI:** 10.3389/fncel.2019.00408

**Published:** 2019-09-09

**Authors:** Ling Xu, Qu Xing, Tuanjie Huang, Jiankang Zhou, Tengfei Liu, Yuanbo Cui, Tian Cheng, Yaping Wang, Xinkui Zhou, Bo Yang, Greta Luyuan Yang, Jiewen Zhang, Xingxing Zang, Shanshan Ma, Fangxia Guan

**Affiliations:** ^1^School of Life Sciences, Zhengzhou University, Zhengzhou, China; ^2^Henan Provincial People's Hospital, Zhengzhou, China; ^3^Translational Medicine Center, Zhengzhou Central Hospital Affiliated to Zhengzhou University, Zhengzhou, China; ^4^The First Affiliated Hospital of Zhengzhou University, Zhengzhou, China; ^5^Stuyvesant High School, New York, NY, United States; ^6^Department of Microbiology and Immunology, Einstein College of Medicine, Bronx, NY, United States

**Keywords:** histone deacetylase 1, human umbilical cord derived mesenchymal stem cells, traumatic brain injury, neuroprotection, PI3K/AKT

In the original article, there was a mistake in Figure 5C as published. The representative propidium iodide (PI) staining photo of the MSC-siHDAC1 group is incorrect. This photo was incorrectly chosen in the process of combining the figure.

**Figure 5:**
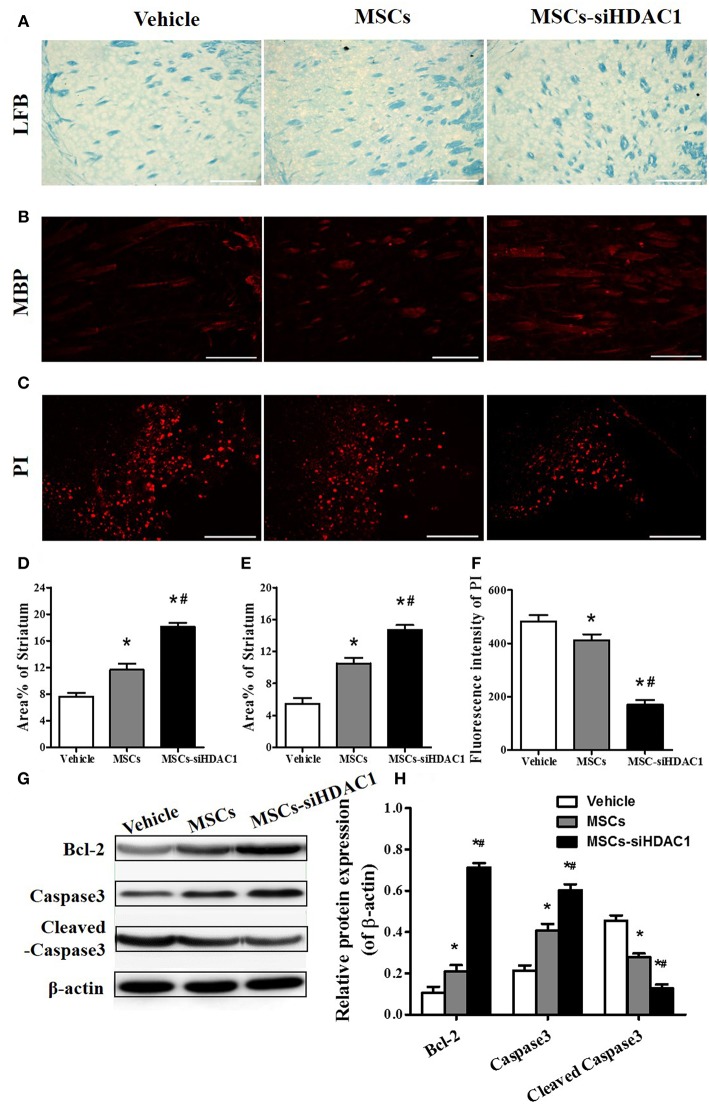
HDAC1-silenced MSCs alleviated white matter injury and reduced cell death after TBI. **(A)** Representative images of luxol fast blue (LFB) staining. Scale bars = 100 μm. **(B)** Myelin basic protein (MBP) staining (red). Scale bars = 200 μm. **(C)** Representative propidium iodide (PI) staining (red) at 3 days after TBI. Scale bars = 100 μm. **(D)** Average area of LFB at 28 days after TBI. **(E)** Average area of MBP. **(F)** Quantitative analysis of PI fluorescence intensity in the injured cortex. **(G)** Western blotting and **(H)** densitometry measurement of Bcl-2, Caspase 3, and Cleaved caspase 3 in the lesion boundary zone of each group at 3 days post-injury. Data are presented as mean ± SEM. ^*^p < 0.05 vs. Vehicle, ^#^*p* < 0.05 vs. MSCs.

At the same time, we also reanalyzed the fluorescence intensity of PI. And, the results showed that MSCs-siHDAC1 more significantly decreased PI fluorescence intensity than that in the original article. The corrected Figure 5 appears below.

The authors apologize for this error and state that this does not change the scientific conclusions of the article in any way. The original article has been updated.

